# A Comparison of Otolaryngology Residency Applicants Over Time and to Other Surgical Applicants

**DOI:** 10.1002/oto2.115

**Published:** 2024-02-22

**Authors:** Matthew E. Lin, Khush Kharidia, Deborah Choe, Neelesh Bagrodia, Neil N. Luu, Tamara Chambers

**Affiliations:** ^1^ Keck School of Medicine of the University of Southern California Los Angeles California USA; ^2^ Department of Internal Medicine University of Texas Southwestern Dallas Texas USA; ^3^ Caruso Department of Otolaryngology–Head and Neck Surgery Keck, School of Medicine of the University of Southern California Los Angeles California USA

**Keywords:** applicant characteristics, residency, surgical subspecialty

## Abstract

**Objective:**

Understand how otolaryngology residency applicant characteristics have changed over time and compare them to those of other surgical subspecialties.

**Study Design:**

Retrospective analysis of academic, extracurricular, and application data in the Texas Seeking Transparency in Application to Residency databases.

**Setting:**

Applicants to otolaryngology, neurological surgery, ophthalmology, plastic surgery, urology, and orthopedic surgery applicants from 2019 to 2023.

**Methods:**

Kruskal‐Wallis, Wilcoxon rank sum, Fischer's exact, and Mann‐Whitney *U* tests were used to compare temporal, match‐based, and subspecialty differences in applicant characteristics.

**Results:**

Across 4 match cycles and 541 otolaryngology applicants, significant differences were found in the average number of honored clerkships per applicant (*P* = 0.044), the percentage of matched applicants (*P* = 0.017), and the average number of research experiences (*P* < 0.001), peer‐revied publications (*P* = 0.002), applied programs (*P* < 0.001), and interviews received (*P* = 0.041). Relative to their unmatched counterparts, matched applicants frequently received more interviews, belonged to higher academic quartiles, and were more likely to belong to academic honor societies (all *P* < 0.05). Matched applicants exhibited significant differences in the number of research experiences (*P* = 0.002), peer‐reviewed publications (*P* = 0.004), and applied programs across cycles (*P* < 0.001). Relative to applicants from other surgical subspecialties, otolaryngology applicants exhibited high amounts of extracurricular involvement, were on par in terms of research output, and received a low proportion of interviews despite applying to a high number of programs.

**Conclusion:**

Matching into otolaryngology has become increasingly competitive and is as competitive as peer surgical subspecialties. Strong academic performance, judicious program signaling, increased research involvement, and holistic factors like letters of recommendation may help applicants successfully match.

With a match rate of 63% in 2021, otolaryngology has become an increasingly selective surgical subspecialty.^1^ From 2019 to 2021, the number of otolaryngology applicants increased by 9.3% annually, while the number of program positions only increased by 3.6%.^1^ Furthermore, otolaryngology has seen a percent increase in applicants over the past several years greater than that of most other specialties.^2^ While these trends make it increasingly important for prospective otolaryngologists to distinguish themselves in the residency match, the elimination of a 3‐digit score for the USMLE Step 1 has made achieving this very feat more difficult by removing a standardized and objective method of cross‐applicant comparison.^3^ While the USMLE Step 2 may be used to achieve the same purpose, it is an imperfect measure, as not all students take Step 2 before applying to residency. As such, less standardized factors such as research, other extracurriculars, and clerkship grades are suggested to become increasingly important in the residency application process.^3^, ^4^, ^5^

Understandably, building a residency application around numerous criteria may contribute to applicant anxiety about applying to a selective specialty like otolaryngology.^1^, ^3^, ^6^ Therefore, it becomes increasingly important to use evidence‐based guidance to inform medical students who are considering otolaryngology as their specialty of choice; by highlighting trends in the qualifications of accepted applicants, prospective applicants can use a data‐driven approach to focus on areas of their application more beneficial to their match. While resources such as the National Resident Matching Program's (NMRP) annual reports on match outcomes provide useful insight into residency applicant characteristics, it can be difficult to parse through its wealth of data to elucidate single‐specialty trends.^2^ Furthermore, while NMRP reports report year‐to‐year changes and allow for cross‐specialty examination, their lack of statistical significance testing limits insight into which of its plethora of reported trends should be emphasized.

As such, one previous study used data from Texas Seeking Transparency in Application to Residency (STAR) database, a nationwide survey administered by the University of Texas Southwestern Medical School to fourth‐year medical students following Match Day, to specifically elucidate the impact of the COVID‐19 pandemic on the otolaryngology match.^7^, ^8^ However, this study did not assess data collected after 2021, a period when the residency application process and the world at large shifted to a period of COVID normalcy. Furthermore, the previous study did not compare otolaryngology applicants to those of other surgical subspecialties, comparisons which may benefit junior medical students as they explore various surgical subspecialties in medical school. As such, we aim to build upon this previous work and use retrospective analysis of data from the Texas (STAR) database to better understand how characteristics of otolaryngology applicants have changed over time and compare to those of applicants in other competitive surgical subspecialties. We hope our findings can provide residency programs and prospective otolaryngology residency applicants with critical guidance as they prepare to navigate through upcoming match cycles.

## Methods

This study was deemed exempt from review by the University of Southern California Institutional Review Board.

This study is a retrospective review of the 2019 to 2023 Texas STAR database, a national annual survey collected by the UT Southwestern Medical School. The deans of student affairs at participating medical schools administer this survey to recently matched fourth‐year medical students in the month following residency match day. In 2023, Texas STAR collected 6962 total responses from 20,384 qualifying medical students from 146 medical schools, resulting in a 34% overall response rate. Previous response rates were 38% in 2022, 40% in 2021, 47% in 2020, and 38% in 2019. Survey responses were included based on application to the following surgical subspecialties with over 100 combined responses in 2022 and 2023: otolaryngology, neurological surgery, ophthalmology, plastic surgery, urology, and orthopedic surgery; otolaryngology data from 2020 and 2021 were also included. Specialty‐specific response rates were unable to be elucidated due to a lack of information on the number of medical students applying to each specialty from participating schools.

The Texas STAR survey reports applicant characteristics akin to what is included in medical students' residency applications. Variables of interest included academic performance (cumulative academic quartile), clerkship honors (number of honors, specialty‐specific honors), honor societies (Alpha Omega Alpha/Sigma, Gold Humanism Honor Society), degrees (MD/DO, other degrees), research (research year, research experiences, abstracts/presentations/posters, peer‐reviewed publications), extracurriculars (volunteer experiences, leadership positions), and successful match status.

Descriptive statistics were utilized to characterize applicants stratified by specialty. Otolaryngology applicants were further stratified by year applied and match status during those years. Kruskal‐Wallis and Fischer's exact tests were used to compare annual differences in applicant characteristics and to compare applicants by surgical subspecialty. Post hoc pairwise comparison was completed using the Wilcoxon rank‐sum test with continuity correction for continuous variables and Bonferroni correction for categorical variables. Fischer's exact and Mann‐Whitney *U* tests were used to compare matched and unmatched applicants. Significance was set at *P* < 0.05. Statistical analysis was performed in Microsoft Excel version 16.70 (Microsoft), R version 4.2.2 (R Foundation), and STATA 18.0 Standard Edition (StataCorp LLC).

## Results

A total of 541 otolaryngology residency applicants were included in the study's cohort. Applicants were predominantly from MD schools (n = 528, 97.60%) and ranked among their medical schools' highest academic quartiles (mean = 1.47, SD = 0.75) (Table 1). Across all 4 years, there was an average match rate of 80.04%.

**Table 1:** Overall Otolaryngology Applicant Characteristics by Year

Category	Overall, n = 541	2019‐2020, n = 143	2020‐2021, n = 115	2021‐2022, n = 137	2022‐2023, n = 146	*P* value^a^
Cumulative quartile, mean (SD)	1.47 (0.75)	1.46 (0.72)	1.47 (0.81)	1.49 (0.76)	1.48 (0.72)	0.960
# Honored clerkships, mean (SD)	3.95 (2.43)	4.36 (2.18)	4.17 (2.36)	3.60 (2.57)	3.71 (2.52)	**0.044**
Honors—this specialty, mean (SD)						
Yes, n (%)	398 (95.90)	114 (95.00)	87 (97.75)	98 (94.23)	99 (97.06)	0.590
AOA/Sigma						
Yes, n (%)	225 (41.59)	53 (37.06)	48 (41.74)	58 (42.34)	66 (45.21)	0.566
GHHS						
Yes, n (%)	78 (14.42)	19 (13.29)	17 (14.78)	22 (16.06)	20 (13.70)	0.917
Degree						
MD, n (%)	528 (97.60)	142 (99.30)	114 (99.13)	132 (96.35)	140 (95.89)	0.129
Other degrees						
Yes, n (%)	101 (21.11)	25 (17.48)	15 (13.04)	28 (20.44)	33 (22.60)	0.094
Research year						
Yes, n (%)	114 (21.11)	26 (18.18)	21 (18.26)	26 (19.11)	41 (28.08)	0.133
# Research experiences, mean (SD)	6.89 (2.91)	6.29 (2.95)	6.57 (2.62)	7.00 (2.73)	7.61 (3.11)	**<0.001**
# Abstracts, presentations, posters, mean (SD)	8.26 (3.32)	7.77 (3.62)	8.00 (3.45)	8.25 (3.15)	8.82 (3.08)	0.0501
# Peer‐reviewed publications, mean (SD)	5.57 (3.46)	4.94 (3.42)	5.20 (3.33)	5.55 (3.27)	6.49 (3.61)	**0.002**
# Volunteer experiences, mean (SD)	7.65 (2.75)	7.79 (2.80)	7.52 (2.73)	7.73 (2.66)	7.55 (2.84)	0.825
# Leadership positions, mean (SD)	5.12 (2.86)	4.91 (2.87)	5.21 (2.92)	4.95 (2.73)	5.43 (2.93)	0.424
# Programs applied, mean (SD)	80.68 (22.37)	73.73 (18.92)	79.94 (18.99)	82.90 (22.92)	86.03 (25.61)	**<0.001**
# Interviews, mean (SD)	12.17 (5.97)	12.08 (5.17)	11.93 (5.54)	11.39 (6.73)	13.18 (6.19)	**0.041**
Matched						
Yes, n (%)	322 (80.04)	124 (86.71)	83 (72.17)	105 (76.64)	121 (82.88)	**0.017**

The bolded *P*‐values are those which are statistically significant (*P* < 0.05).

Abbreviations: AOA, Alpha Omega Alpha; GHHS, Gold Humanism Honor Society.

^a^ *P* values correspond to significant differences across all 4 match cycles and do not include the overall applicant column in its assessment.

Table 1 also depicts otolaryngology applicant characteristics by cycle. Across the 4 cycles measured, significant differences were found in the average number of honored clerkships per applicant (*P* = 0.044) and percentage of matched applicants (*P* = 0.017), both of which exhibited decreases over the 4 cycles. The average number of applicant research experiences (*P* < 0.001) and peer‐reviewed publications (*P* = 0.002) also significantly differed across application cycles; both exhibited an overall increase over time (research experiences: 6.29 in 2019 to 2020 cycle vs 7.61 in 2022 to 2023 cycle; peer‐reviewed publications: 4.94 in 2019 to 2020 cycle vs 6.49 in 2022 to 2023 cycle). Similarly, the number of average programs applied to and interviews received significantly differed between cycles, with an overall increase exhibited (number of programs applied: 73.73 in 2019 to 2023 cycle vs 86.03 in 2022 to 2023 cycle; the number of interviews: 12.08 in 2019 to 2020 cycle vs 13.18 in 2022 to 2023 cycle). No other significant differences were observed.

Otolaryngology applicant characteristics stratified by match status (matched or not matched) are detailed in Table 2. Matched applicants consistently belonged to higher academic quartiles relative to their unmatched counterparts (2019‐2020 cycle *P* = 0.009, 2021‐2022 cycle *P* = 0.0496, 2022‐2023 cycle *P* = 0.007). Matched applicants were also significantly more likely to belong to the AOA or Sigma academic honor societies in the 2019 to 2020 (*P* = 0.044) and 2021 to 2022 (*P* = 0.026) match cycles. Whereas matched applicants reported significantly more abstracts/presentations/posters (*P* = 0.004), peer‐reviewed publications (*P* = 0.007), and leadership positions (*P* = 0.012) relative to their unmatched peers in the 2019 to 2020 match cycle, this trend did not persist in subsequent cycles. Across all 4 match cycles, matched applicants consistently received more interviews than did unmatched applicants (*P* < 0.001 for all 4 cycles).

**Table 2:** Otolaryngology Applicant Characteristics by Match Status and Year

	2019‐2020			2020‐2021			2021‐2022			2022‐2023			Matched	Nonmatched
Category	Matched, n = 124	Nonmatched, n = 19	*P* value	Matched, n = 83	Nonmatched, n = 32	*P* value	Matched, n = 105	Nonmatched, n = 32	*P* value	Matched n = 121	Nonmatched n = 25	*P* value	*P* value^a^	*P* value
Cumulative quartile, mean (SD)	1.38 (0.65)	1.94 (0.93)	**0.009**	1.43 (0.78)	1.57 (0.90)	0.545	1.39 (0.66)	1.82 (0.96)	**0.0496**	1.39 (0.67)	2.00 (0.82)	**0.007**	1.000	0.386
# Honored clerkships, mean (SD)	4.45 (2.16)	3.74 (2.26)	0.157	4.25 (2.19)	3.97 (2.80)	0.657	3.70 (2.61)	3.28 (2.45)	0.423	3.69 (2.61)	3.80 (2.08)	1.000	0.069	0.691
Honors—this specialty														
Yes, n (%)	99 (96.11)	15 (88.24)	0.201	63 (98.44)	24 (96.00)	0.485	75 (96.15)	23 (88.46)	0.163	83 (97.65)	16 (94.11)	0.425	0.830	0.819
AOA/Sigma														
Yes, n (%)	50 (40.32)	3 (15.79)	**0.044**	38 (45.78)	10 (31.25)	0.206	50 (47.62)	8 (25.00)	**0.026**	56 (48.28)	10 (40.00)	0.661	0.687	0.342
GHHS														
Yes, n (%)	18 (14.52)	1 (5.26)	0.469	30 (36.14)	9 (28.13)	1.000	17 (16.19)	5 (15.63)	1.000	15 (12.40)	5 (20.00)	0.340	0.873	0.634
Degree														
MD, n (%)	123 (99.19)	19 (100.00)	1.000	82 (98.80)	32 (100)	1.000	102 (97.14)	30 (93.75)	0.322	115 (95.04)	25 (100.00)	0.590	0.203	0.335
Other degrees														
Yes, n (%)	20 (16.13)	5 (26.32)	0.775	12 (14.46)	3 (9.38)	0.242	18 (17.14)	10 (31.25)	0.600	22 (18.18)	11 (44.00)	0.680	0.095	0.871
Research year														
Yes, n (%)	23 (18.55)	3 (15.79)	1.000	17 (20.48)	4 (12.50)	0.424	19 (18.10)	7 (22.58)	0.607	33 (27.27)	8 (32.00)	0.631	0.302	0.338
# Research experiences, mean (SD)	6.42 (2.91)	5.42 (3.17)	0.186	6.75 (2.35)	6.13 (3.22)	0.428	7.01 (2.72)	6.97 (2.78)	0.935	7.70 (2.95)	7.16 (3.82)	0.673	**0.002**	0.273
# Abstracts, presentations, posters, mean (SD)	8.14 (3.43)	5.28 (4.00)	**0.004**	8.39 (3.16)	6.93 (4.00)	0.057	8.54 (2.99)	7.88 (3.22)	0.263	9.04 (2.95)	7.71 (3.52)	0.075	0.186	0.133
# Peer‐reviewed publications, mean (SD)	5.19 (3.36)	3.00 (3.33)	**0.007**	5.39 (3.41)	4.72 (3.10)	0.399	5.73 (3.19)	4.94 (3.49)	0.168	6.74 (3.54)	5.32 (3.78)	0.089	**0.004**	0.115
# Volunteer experiences, mean (SD)	7.82 (2.73)	7.56 (3.36)	0.856	7.55 (2.79)	7.44 (2.58)	0.757	7.66 (2.65)	7.94 (2.71)	0.686	7.69 (2.82)	6.83 (2.84)	0.174	0.940	0.623
# Leadership positions, mean (SD)	5.17 (2.89)	3.28 (2.08)	**0.012**	5.38 (3.07)	4.78 (2.46)	0.401	4.81 (2.69)	5.43 (2.86)	0.338	5.65 (2.92)	4.30 (2.80)	0.085	0.230	0.088
# Programs applied, mean (SD)	73.42 (19.47)	75.79 (15.12)	0.755	80.65 (17.92)	78.09 (21.72)	0.788	82.08 (24.14)	85.68 (18.27)	0.557	86.59 (24.46)	83.32 (31.05)	0.835	**<0.001**	0.173
# Interviews, mean (SD)	12.75 (4.83)	7.74 (5.30)	**<0.001**	13.23 (5.25)	8.56 (4.89)	**<0.001**	12.29 (6.35)	8.47 (7.20)	**<0.001**	13.98 (5.76)	9.32 (6.88)	**<0.001**	0.060	0.697

The bolded *P*‐values are those which are statistically significant (*P* < 0.05).

Abbreviations: AOA, Alpha Omega Alpha; GHHS, Gold Humanism Honor Society.

^a^ Matched and nonmatched *P* value columns correspond to significant differences among applicants across all four application cycles, stratified by match status. All other *P* values correspond to differences between matched and nonmatched applicants from a single applicant cycle.

Across all four match cycles, significant differences in the number of research experiences (*P* = 0.002) and peer‐reviewed publications (*P* = 0.004) existed among matched applicants, with both characteristics exhibiting increases over time (research experiences: 6.42 in the 2019‐2020 cycle vs 7.70 in the 2022‐2023 cycle; peer‐reviewed publications: 5.19 in the 2019‐2020 cycle vs 6.74 in the 2022‐2023 cycle). Furthermore, matched applicants also exhibited significant differences in the number of programs applied across cycles (*P* < 0.001) with a similar upward trend (73.42 in the 2019‐2020 cycle vs 86.59 in the 2022‐2023 cycle). No characteristic significantly differed between application cycles among non‐matched applicants.

In the 2021 to 2022 and 2022 to 2023 application cycles, there were 382 otolaryngology applicants, 136 neurological surgery applicants, 306 ophthalmology applicants, 114 plastic surgery applicants, 244 urology applicants, and 572 orthopedic surgery applicants (Table 3). Applicant characteristics stratified by subspecialty are also presented in Table 3.

**Table 3:** Otolaryngology Applicant Characteristics Relative to Those of Other Competitive Surgical Specialties (2021‐2022 and 2022‐2023 Cycles)

	Otolaryngology, n = 283	Neurological surgery, n = 136	Ophthalmology, n = 306	Plastic surgery, n = 114	Urologic surgery, n = 244	Orthopedic surgery, n = 572	*P* value
Cumulative quartile, mean (SD)	1.48 (0.74)	1.44 (0.72)	1.49 (0.77)	1.43 (0.72)	1.61 (0.82)	1.54 (0.83)	0.479
# Honored clerkships, mean (SD)	3.66 (2.54)	3.83 (1.68)	3.76 (2.60)	4.00 (2.57)	3.43 (2.66)	3.83 (2.55)	0.332
Honors—this specialty, mean (SD)							**0.049**
Yes, n (%)	197 (95.63)	103 (97.17)	187 (97.40)	93 (97.89)	185 (92.50)	397 (92.76)	
AOA/Sigma							0.067
Yes, n (%)	124 (43.82)	50 (36.76)	133 (36.94)	49 (42.98)	79 (32.38)	226 (39.51)	
GHHS							0.553
Yes, n (%)	42 (14.84)	18 (13.24)	55 (17.98)	14 (12.28)	34 (13.93)	77 (13.46)	
Degree							**<0.001**
MD, n (%)	272 (96.11)	136 (100.00)	300 (98.04)	113 (99.12)	240 (98.36)	534 (93.36)	
Other degrees							**<0.001**
Yes, n (%)	61 (21.55)	46 (33.82)	57 (18.63)	24 (21.05)	56 (22.95)	99 (17.31)	
Research year							**<0.001**
Yes, n (%)	67 (23.76)	31 (22.96)	59 (19.41)	33 (28.95)	27 (11.07)	89 (15.59)	
# Research experiences, mean (SD)	7.31 (2.94)	6.71 (2.73)	5.92 (2.99)	7.31 (3.14)	6.38 (3.08)	6.16 (2.97)	**<0.001**
# Abstracts, presentations, posters, mean (SD)	5.82 (2.83)	5.29 (2.60)	6.59 (2.93)	5.66 (3.00)	6.47 (2.95)	5.86 (2.82)	**<0.001**
# Peer‐reviewed publications, mean (SD)	7.04 (3.47)	8.29 (3.65)	5.55 (3.36)	7.95 (3.68)	5.52 (3.36)	6.15 (3.88)	**<0.001**
# Volunteer experiences, mean (SD)	8.63 (2.75)	7.29 (2.96)	6.72 (2.68)	8.10 (2.85)	7.29 (3.05)	8.34 (2.93)	**<0.001**
# Leadership positions, mean (SD)	6.20 (2.84)	5.68 (2.78)	5.32 (2.22)	6.22 (2.78)	5.32 (2.74)	5.82 (2.65)	**<0.001**
# Programs applied, mean (SD)	71.02 (22.83)	66.84 (23.91)	66.47 (21.57)	64.51 (15.70)	67.25 (24.56)	73.25 (30.31)	**<0.001**
# Interviews, % (SD)	13.30 (6.47)	18.89 (7.96)	11.40 (4.53)	13.64 (6.51)	14.61 (5.80)	12.20 (5.71)	**<0.001**
# Interviews from applied programs, % (SD)^a^	17.34 (15.68)	25.31 (15.21)	15.10 (12.34)	22.87 (60.69)	25.21 (96.68)	16.79 (46.75)	**<0.001**
Matched							
Yes (n, %)	226 (79.86)	119 (87.50)	262 (86.18)	78 (68.42)	208 (85.25)	475 (83.04)	**<0.001**

The bolded *P*‐values are those which are statistically significant (*P* < 0.05).

Abbreviations: AOA, Alpha Omega Alpha; GHHS, Gold Humanism Honor Society.

^a^ Interviews from applied programs are calculated by dividing the number of interviews received by the number of programs applied.

Whereas significant differences across surgical subspecialties were found among most characteristics, Table 4 details a post hoc analysis assessing differences between individual surgical subspecialties compared to otolaryngology. Otolaryngology applicants reported a higher number of research experiences (mean = 7.31, SD = 2.94) that was significantly higher than that of applicants to ophthalmology (*P* < 0.001), urologic surgery (*P* = 0.001), and orthopedic surgery (*P* < 0.001). However, they reported significantly fewer abstracts, presentations, and posters (mean = 5.82, SD = 2.83) relative to ophthalmology (*P* = 0.001) and urologic surgery (*P* = 0.004) applicants. Otolaryngology applicants also reported significantly fewer publications (mean = 7.04, SD = 3.47) relative to their counterparts in neurologic surgery (*P* = 0.001) and plastic surgery (*P* = 0.031), although they reported significantly more publications than applicants to ophthalmology (*P* < 0.001), urologic surgery (*P* < 0.001), and orthopedic surgery (*P* < 0.001). Otolaryngology applicants were also highly involved in other extracurriculars, reporting significantly more leadership positions (mean = 6.20, SD = 2.84) and volunteer experiences (mean = 8.64, SD = 2.75) than ophthalmology (both *P* < 0.01) and urologic surgery (both *P* < 0.01) applicants.

**Table 4:** Post Hoc Subgroup Analysis of Otolaryngology Applicant Characteristics Relative to Those of Other Competitive Surgical Specialties (2021‐2022 and 2022‐2023 cycles)^a^, ^b^, ^c^, ^d^

Avg (SD), *P* value	Otolaryngology	Neurological surgery	Ophthalmology	Plastic surgery	Urologic surgery	Orthopedic surgery
Research year; n (%), *P* value	67 (23.76)		‐	‐	27 (11.07), *P* = 0.003	‐
# Research experiences	7.31 (2.94)	‐	5.92 (2.99), *P* < 0.001	‐	6.38 (3.08), *P* = 0.001	6.16 (2.97), *P* < 0.001
# Abstracts, presentations, posters	5.82 (2.83)	‐	6.59 (2.93), *P* = 0.001	‐	6.47 (2.95), *P* = 0.004	‐
# Peer‐reviewed publications	7.04 (3.47)	8.29 (3.65), *P* = 0.001	5.55 (3.36), *P* < 0.001	7.95 (3.68), p = 0.031	5.52 (3.36), *P* < 0.001	6.15 (3.88), *P* < 0.001
# Volunteer experiences	8.63 (2.75)	7.29 (2.96), *P* < 0.001	6.72 (2.68), *P* < 0.001	‐	7.29 (3.05), *P* < 0.001	‐
# Leadership positions	6.20 (2.84)	‐	5.32 (2.22), *P* = 0.004	‐	5.32 (2.74), *P* = 0.004	‐
# Programs applied	71.02 (22.83)	‐	66.47 (21.57), *P* = 0.021	64.51 (15.70), p = 0.009	67.25 (24.56), *P* = 0.032	‐
# Interviews	13.30 (6.47)	18.89 (7.96), *P* < 0.001	11.40 (4.53), *P* = 0.002	‐	14.61 (5.80), *P* = 0.012	12.20 (5.71), *P* = 0.026
% Interviews from applied programs; n (%), *P* value	17.34 (15.68)	25.31 (15.21), *P* < 0.001	‐	‐	25.21 (96.68), *P* = 0.002	16.79 (46.75), *P* = 0.025

^a^ Values listed correspond to post hoc *P* value.

^b^ Only significant differences are presented to reduce table complexity.

^c^ Green denotes otolaryngology significantly higher than other surgical subspecialties on post hoc analysis, red denotes otolaryngology significantly lower than other surgical subspecialties on post hoc analysis, and white denotes no significant difference between otolaryngology and other surgical subspecialties. This pattern was reversed for the number of interviews received and the percentage of interviews from programs applied.

^d^ Post hoc pairwise comparison of continuous variables completed through Wilcoxon rank‐sum test with continuity correction. Post hoc pairwise comparison of categorial variables completed through Bonferroni correction.

Otolaryngology applicants applied to a higher number of residency programs (mean = 71.02, SD = 22.83), significantly higher than what was reported by applicants to ophthalmology (*P* = 0.021), plastic surgery (*P* = 0.009), and urologic surgery (*P* = 0.002). Relative to other surgical subspecialty applicants, however, otolaryngology applicants received a low proportion of interviews from the programs they applied to (mean = 17.34%, SD = 15.68%). Although they received a higher proportion than did orthopedic surgery applicants (*P* = 0.025), they received a significantly lower proportion of interviews than their peers applying for neurological surgery (*P* < 0.001) and urologic surgery (*P* = 0.002).

## Discussion

Securing a post‐graduate residency training position in an otolaryngology program is a dynamic and complex process. Over the past four years, the otolaryngology match rate has been significantly different, showing a downward trend. During the 2019 to 2020 application cycle, there was a significant difference in the academic quartiles, research productivity, and leadership positions between matched and unmatched applicants. There were several notable patterns for otolaryngology applicants over the four application cycles, including a trend toward more research among all applicants and more total applications submitted among matched applicants. One predictor of matching into otolaryngology that remained consistent over the years was the number of interviews received. Applicants to otolaryngology were very highly qualified in comparison with those of other competitive surgical subspecialties but received a small proportion of interviews relative to peer subspecialty applicants.

Only one previous study has used the Texas STAR database to assess the impact of the COVID‐19 pandemic on the otolaryngology match by comparing applicants from 2018 to 2020 to those from 2021.^7^ While it found both matched and unmatched applicants endorsed significantly more publications during the 2020 to 2021 COVID application cycle relative to applicants of previous years, we found significant differences in the number of applications among matched applicants but not among unmatched applicants. This difference may stem from the time periods studied: while it is understandable that all applicants would apply with more research to showcase interest in otolaryngology and maintain exposure to the field during a period of social isolation and limited away rotation opportunities, this persistent increase in research output among matched applicants may suggest continued research productivity may still be beneficial to successfully matching into otolaryngology.

While unmatched applicants reported significantly lower research output and leadership experiences relative to matched applicants in the 2019 to 2020 match cycle, these differences did not persist in subsequent cycles. This suggests extracurricular involvement may no longer be a key differentiating factor between matched and unmatched applicants. Additionally, the lack of significant differences in the percentage of respondents who took a research year or had other degrees between matched and unmatched applicants questions the role of the research year. While research years or dual degrees may help students explore otolaryngology and network within the field, they are not necessary for students who have been able to engage in productive otolaryngology scholarship throughout medical school.

Across multiple cycles, matched applicants belonged to significantly higher academic quartiles and were more likely to belong to national academic honor societies. Although these measures are certainly imperfect given the subjective and highly variable nature of clerkship grading and efforts nationwide to cut ties with the Alpha Omega Alpha honor society in light of potential racial bias in its member selection, these measures can still provide some level of insight on how otolaryngology applicants perform relative to their peers over a period of several years, and as such could be suggested as areas of focus for prospective otolaryngologists going forward.^9^, ^10^

One trend that remained consistently different between matched and unmatched applicants was in the number of interviews received. While this finding was expected, this difference may stem from the number of programs applicants applied to. Although both matched and unmatched applicants demonstrated upward trends in the number of applications submitted per applicant, these differences between cycles were only significant among matched applicants. This trend is also reflected in our overall cohort, as both the average number of applied programs and interviews per applicant increased and were significantly different across 4 cycles. While our finding corroborates previous studies advocating the benefits of applying broadly for the otolaryngology match, it is unclear how changes to the otolaryngology signaling process will affect the suggestion to broadly apply.^6^

Although prospective otolaryngologists applied to the second‐most number of programs across all 6 surgical subspecialties, they received the third‐fewest interviews; these findings are corroborated by significance testing across cohorts and post‐hoc pairwise comparisons. While these trends may also change following adjustments to the otolaryngology match signaling system, this outcome still attests to how competitive matching into otolaryngology is.

Otolaryngology applicants were expectedly found to largely be on par with other surgical subspecialty applicants. Many characteristics exhibited no significant differences across subspecialties, and otolaryngology applicants reported significantly more research experiences, leadership positions, and volunteer experiences than applicants to multiple other fields. Although otolaryngology applicant research output in the form of posters, presentations, abstracts, and papers were both significantly higher and lower than that of other subspecialties, our other findings suggesting the potential benefits of increased research to matching into otolaryngology indicate similar advice regarding high research involvement should be given regardless of subspecialty interest. Based on our findings, potential otolaryngologists considering the field among other surgical specialties may benefit by heeding advice geared towards a successful match in otolaryngology, as doing so will place them on par, if not ahead, of their other subspecialty peers and position them well to apply into any specialty of their choosing.

The switch to a pass‐fail grading system of USMLE Step 1 means that greater emphasis is now placed on other components of an applicant's resume, although the distribution of factor importance is not yet fully understood. On top of that, the increasing selectiveness of otolaryngology and the narrowing gap between matched and unmatched applicants beg the question: What can applicants do to stand out amongst a highly qualified applicant pool to secure a coveted otolaryngology residency spot?

Based on our findings, we offer three recommendations. First, we suggest continuing to showcase academic excellence through institution‐specific metrics and national honor societies: although imperfect, they remain the best metric available for applicants to showcase their intellectual and clinical abilities relative to their peers.

Second, we encourage students to seek out research opportunities early on during medical school to reduce the pressure to pursue a research year and maximize the time they have available to submit or publish their work before submitting their residency applications. Early research involvement also gives students more time to explore the field of otolaryngology, connect with faculty, and deepen relationships with mentors. In turn, these longitudinal experiences can strengthen letters of recommendation and personal statements while also bolstering applicants' ability to speak intelligibly about the field during interviews—all subjective factors that have become increasingly emphasized over time.^5^, ^11^, ^12^

Finally, we advise applicants to judiciously select the programs they signal as otolaryngology moves to high‐volume signaling. While signaling a program may significantly increase the likelihood of an interview invite, some programs will receive more signals than others, and many may receive more signals than interview spots available.^13^ As we found matched applicants endorsed significantly more interviews than their unmatched peers across all application cycles, it may be beneficial for applicants’ signal lists to primarily consist of programs that both fit their personal preferences and are likely to extend an interview offer. Doing so would provide applicants more opportunities to showcase their merits during interviews and rank more programs, which has been previously suggested to increase the likelihood of matching into residency.^2^

The results of the study should be interpreted cautiously as the use of the Texas STAR database limits the generalizability of the results to all otolaryngology applicants. The survey completion rates of 38%, 47%, 40%, 38%, and 34% in the years from 2019 to 2023, respectively, do not necessarily capture representative experiences from all applicants.^8^ The survey captured data from very few DO students and no international medical graduates who together made up 17% of all otolaryngology applicants in 2022, thus limiting generalizability to US MD students.^14^ Board scores were reported within 5‐point ranges, which prevented the calculation of standard deviation and significant differences beyond the average board scores provided for each cohort. Previous TEXAS STAR analysis by Lenze et al revealed a geographic bias to the South as the database initiative originated at the University of Texas Southwestern. Though more medical schools are now participating, this geographic bias limits generalizability.^7^ As such, we were unable to assess temporal changes in the number of applicants and applicants per position using this database.

This analysis is also subject to selection bias. Matched otolaryngology applicants were overrepresented in the analysis. As unmatched applicants were less likely to respond to the survey, the data from unmatched applicants should be interpreted with caution. Despite its limitations, the data from Texas STAR provides transparency by reporting individual data that other databases like NRMP or FREIDA do not.^7^ Future studies could investigate the role of demographic factors, medical school ranking, and preference signaling in the selection process. Additionally, these studies could provide additional insight into how the weightage of resident selection factors changes when evaluating applicants without a scored USMLE Step 1 result.

## Conclusion

Matching into otolaryngology has become more competitive over time, especially considering diminishing differences between matched and unmatched applicants. Relative to medical students applying to other surgical subspecialties, otolaryngology applicants remain among the most qualified. While demonstrating a strong resume remains key to matching, increased research involvement, judicious program signaling, and holistic factors such as letters of recommendation, personal statements, and demonstrated interest in otolaryngology may also be beneficial to ensure a successful match.

## Author Contributions

**Matthew E. Lin**, conception and design of work, data acquisition and analysis, interpretation of data, drafting of manuscript, critical revision; **Khush Kharidia**, interpretation of data, drafting of manuscript, critical revision; **Deborah Choe**, drafting of manuscript, critical revision; **Neelesh Bagrodia**, data acquisition and analysis, critical revision; **Neil N. Luu**, conception and design of work, interpretation of data, critical revision; **Tamara Chambers**, conception and design of work, interpretation of data, critical revision.

## Disclosures

### Competing interests

None.

### Funding source

None.
